# THE PHYSICAL AND MENTAL HEALTH OF HOME HEALTHCARE WORKERS: WHAT DO WE KNOW AND WHAT CAN WE DO ABOUT IT?

**DOI:** 10.1093/geroni/igac059.626

**Published:** 2022-12-20

**Authors:** Madeline Sterling

**Affiliations:** Weill Cornell Medicine, New York City, New York, United States

## Abstract

This presentation will use data from the 2014-2018 CDC's Behavioral Risk Factor Surveillance Survey to characterize the physical and mental health of home health care workers compared to other similar frontline, low-wage worker groups, before the COVID-19 pandemic. Next, using primary survey data collected in partnership with the 1199SEIU Training and Employment Fund, the health of the workforce during the COVID-19 pandemic will be presented, as well as the workforces' needs. The survey includes data from over 250 home health care workers employed in New York, NY who provided care during the pandemic. Finally, data from a pilot feasibility study of an intervention aimed at improving home care workers' well-being will be presented.

